# Strengthening regional preparedness and surge workforce through the Global Outbreak Alert and Response Network

**DOI:** 10.5365/wpsar.2024.15.5.1353

**Published:** 2024-11-17

**Authors:** Sharon Salmon

**Affiliations:** a World Health Organization Regional Office for the Western Pacific, Manila, Philippines.; b UNSW Medicine & Health, School of Population Health, University of New South Wales, Sydney, New South Wales, Australia.

Public health emergencies continue to pose significant threats to global health security. Whether due to novel pathogens or climate issues, the frequency, scale and complexity of outbreaks are increasing. No single institution or country has the capacity to address these threats alone. Coordinated, multidisciplinary and international mechanisms like the Global Outbreak Alert and Response Network (GOARN) are now more essential than ever.

Since the World Health Organization (WHO) established GOARN in 2000, the network has provided a trusted and reliable mechanism to rapidly mobilize technical assistance to countries facing public health threats. GOARN is an integral component of the broader emergency workforce architecture, complementing emergency medical teams and contributing to the Global Health Emergency Corps framework, (*1*) which collectively strengthen readiness and response capacities worldwide. With more than 320 partners worldwide, (*2*) GOARN identifies and deploys experts during health emergencies, while also strengthening public health capacities between crises. In the WHO Western Pacific Region, where countries face diverse geographic and health system contexts, GOARN has helped fill critical technical human resource gaps at times when countries need support most.

This special edition provides insights into the response operations, technical assistance provided and GOARN partner initiatives. (*3*) It showcases the diversity of partner engagement in the Region and highlights areas for future investment including gender equity in outbreak leadership (*4*) and strategic partner contributions. (*5*)

A prominent theme is the importance of a ready-to-respond and agile emergency workforce. GOARN continues to demonstrate its lead role in operationalizing this by serving as both a surge deployment mechanism and an emergency workforce capacity-strengthening platform, linking global expertise with national needs.

The analysis of GOARN deployments during the COVID-19 pandemic further enforces the critical role of international surge support in strengthening health systems during a global public health emergency. (*6*) Complementing this, the first regional analysis of GOARN deployments to, from and within the WHO Western Pacific Region provided a 24-year review, revealing the breadth of partner engagement and the need to continue to engage more partners to contribute to the surge workforce. (*7*) Together, these analyses demonstrate that GOARN is not only a response mechanism but also a strategic enabler of sustainable public health capacity.

Whether through deployment of epidemiologists, laboratory specialists or infection prevention and control experts, GOARN supports countries to scale up their response when local capacities are overwhelmed. At the same time, by embedding experts within national systems, each deployment becomes an opportunity to transfer knowledge, mentor local responders and build sustainable public health capabilities.

Looking ahead, strengthening the health emergency workforce must remain a top national priority. This means not only maintaining the capacity to deploy experts rapidly during crises but also investing in workforce preparedness between emergencies. GOARN’s unique position at the intersection of response and capacity-strengthening makes it an essential pillar in this effort.

This special edition serves as both a reflection on past achievements and a call to action, to continue investing in the people, systems and partnerships that make effective emergency response possible, locally, regionally and globally.
